# Early Rising during the Puerperium

**Published:** 1909-10-30

**Authors:** 


### Gynæcology.

### EARLY RISING DURING THE PUERPERIUM.

The time-honoured method of keeping the puerperal woman in bed for at least ten days has been seriously questioned, and rising at a much earlier date has been extensively recommended and tried. This principle too has been extended to the treatment of abdominal sections, and we read of patients being encouraged to get up out of bed on the third day after abdominal hysterectomy and to walk about as early as the fifth day. The chief reasons advanced for this treatment of the puerperal state are briefly that early rising assists involution of the uterus, that it prevents backward displacements, and that it prevents thrombosis and consequently diminishes the danger of pulmonary embolism. On the other hand, certain contraindications to early rising are admitted on all hands; particularly septic infections, difficult labour with extensive injuries to the soft parts, and post-partum hæmorrhage. Furthermore, the social status of the patient cannot be ignored, for it must not be thought that a patient is ready to work hard because she is permitted to get up earlier than formerly, and it is a matter of common experience amongst the poorer sections of the community that the mother begins to work as soon as she is permitted to get up from child-bed. The results of the new methods have not been uniformly good, and a case reported by Frommer (*Zentralb. f. Gyn.* 1909, No. 1) shows that early rising will not prevent pulmonary embolism or thrombosis in a septic case. This case, however, is hardly fair criticism, for the patient only got up for one hour on the second day, and on the following day developed symptoms of septic infection. Although the fever lasted only three days, she died with an embolism on the eleventh day: thrombosis of the left ovarian vein was found post-mortem. We cannot see that the early rising could have contributed to the fatal result in this case.

To gauge the value of our old methods as compared with the new we must dispassionately review our past experience, and the final results of the new methods cannot be truly criticised until we know something of the later history of these patients. A low morbidity percentage in hospital is no guarantee that there will be no subinvolution later, with the attendant evils of uterine congestion and endometritis. We want to know what the health of these patients is a year after labour. In the past it has been the custom to keep patients lying in the supine position for at least a week, and whether the doctor has insisted on this or not, it has been the posture naturally assumed by the patient. Also the patient has been encouraged to remain as still as possible, and not to exert herself to move. This no doubt is going too far in the other direction, for immobility tends to reduce the circulation rate and the force of the heart beat, and can only favour thrombosis in those disposed to it. Further, the dorsal position is positively the worst for drainage of the uterus (the Trendelenberg position only excepted). This must favour sepsis by supplying stagnant fluids for the development of bacteria, and must prevent natural involution to some extent. Finally, the dorsal position is positively proved to be the prime cause of retroversion and flexion of the uterus in by far the larger proportion of such cases. It is difficult to conceive of a definitely proved acquired retroversion and flexion of the uterus in a person who has never been pregnant. We cannot say that such cases never occur, but when we meet cases of retroversion and flexion in virgins it is practically impossible to prove a cause for them and to say that they are not congenital or at least acquired in early infancy. It may therefore be confidently assumed that the patient who is permitted to lie on her side or semiprone will drain better, will involute better, and will not run the risk of acquiring a backward displacement.

What, then, is the risk to the patient if permitted to sit up in bed, or out of bed, soon after delivery? Is the risk of thrombosis or embolism increased? Theoretically, it should not be, because in a normal labour with a normally contracted and retracted uterus there are no obvious thrombi in the vessels at the placental site. These vessels are closed by muscle tonus and their open ends heal with a minimum amount of coagulation. Is sitting likely to increase this coagulation? Theoretically no, because the rapidity of the general circulation is thereby increased, and the more rapid the circulatory rate the less stasis, even in severed vessels, and the less likelihood of coagulation. If, however, the puerperal conditions are not normal, and any pathological condition favouring blood coagulation is present, then the movements entailed in sitting up will certainly increase the risk of embolism. What experience have we in this country of early rising after labour? Very little, but what there is certainly provides arguments against, instead of for, the practice. It is the common complaint of the Masters of the Rotunda Hospital that they cannot keep their patients long enough in bed. This means that the patients get up, go home on their own responsibility, and commence their usual avocations. Are the results of this practice favourable? We judge that they are not, if the literature of accidental hæmorrhage is considered. The only work worth considering on accidental hæmorrhage in this country of late years has been done at the Rotunda Hospital, and it could not have been done unless the material had been there. Accidental hæmorrhage is predisposed to by endometritis, subinvolution, and uterine congestion generally, and we believe that the comparatively large amount of material available at the Rotunda Hospital in this respect is connected with the habits of early rising after labour plus immediate resumption of hard work, in spite of medical advice to the contrary. There is no literature worth considering from the London lying-in hospitals on accidental hæmorrhage, and it is the custom of the poorest in London to remain in bed nine days after delivery. We believe the two are intimately connected. To sum up these arguments, then, we feel that early rising in normal labours cannot do much harm, and may be productive of some good results; but early rising must not mean an earlier resumption of work.

